# Nifedipine compared to magnesium sulfate for treating preterm labor: A randomized clinical trial

**Published:** 2014-02

**Authors:** Roshan Nikbakht, Mahin Taheri Moghadam, Homa Ghane’ee

**Affiliations:** 1 *Department of Obstetrics and Gynecology, Ahvaz Jondishapour University of Medical Sciences, Ahvaz, Iran.*; 2 *Department of Pathology, Ahvaz Jondishapour University of Medical Sciences, Ahvaz, Iran.*

**Keywords:** *Nifedipine*, *Magnesium sulfate*, *Preterm labor*

## Abstract

**Background:** Preterm labor is the leading cause of infant morbidity and mortality so it may be necessary to administer tocolytics for treatment of it.

**Objective: **The aim of this study was to compare the efficacy and safety of magnesium sulfate and nifedipine in the management of preterm labor.

**Materials and Methods:** 100 women with documented preterm labor were randomly assigned to receive magnesium sulfate (n=50) and nifedipine (n=50) as tocolytic therapy. Before tocolysis, patient did not receive any sedation. After tocolysis, if patient continued to have contractions, they received other tocolytic agents. The main outcome variables examined were days gain in utero, success rate and side effects of tocolysis.

**Results:** Both drugs were equally effective in prevention of labor and delaying delivery >7 days, 56% vs. 64% in the nifedipine and magnesium sulfate groups, and the days gain in utero was no statistically different in two groups. 6% of nifedipine group and 2% of magnesium sulfate group required drug discontinuation due to severe symptoms. There were also no significant differences in maternal characteristics between two groups. The total success rate and side effects were similar in two groups.

**Conclusion:** Oral nifedipine could be a suitable alternative for magnesium sulfate with the same efficacy and side effects in the management of preterm labor.

**Registration ID in IRCT:** IRCT2013090914603N1

## Introduction

Preterm labor is frequency uterine contractions, progressive effacement and dilation of the cervix prior to term gestation. Prevention and treatment of preterm labor is important because it is one of the most important causes of perinatal morbidity and mortality    (1) . The etiology of preterm labor is poorly understood. The maternal risk factors include previous abortions, previous preterm labor, genital and urinary tract infection    (2) . Preterm infants may have developmental delay, visual and hearing impairments, neurological problems or lung disease    (3) . 

A wide range of tocolytics are used to suppress uterine contractions. Magnesium sulfate is used in many centers (4). Randomized trial revealed that combination therapy, magnesium with ritodrine, significantly reduced uterine contractions rather than changing into magnesium alone (5). Moreover, the currently available data reveals that magnesium sulfate tocolysis does not reduce the frequencies of delivery and is not associated with improvements in newborn morbidities or mortality (6). A Cochrane systematic review showed that magnesium sulfate is ineffective as a tocolytic (7). Many obstetricians use nifedipine as a tocolytic (8). 

Some studies showed that nifedipine in comparison with other tocolytics is associated with a more frequent successful prolongation of pregnancy and lower incidence of respiratory distress syndrome (9). Conde-Agudelo *et al* studied 26 trials (2179 women) and showed that nifedipine had a significant reduction in the risk of delivery within 7 days of initiation of treatment and before 34 weeks’ gestation (10). In another study, Lyell and colleges compared magnesium sulfate with nifedipine and they conclude that two tocolytics were similar in delay of delivery, gestational age at delivery, and neonatal outcomes (11).

The objective of our study is to compare the effectiveness of magnesium sulfate and nifedipine (Adalat) in the management of patients admitted with the diagnosis of preterm labor. In addition, our study will try to assess the safety of these tocolytics on the mother by assessing a selected number of outcome variables. The data will be used to change our protocol for management of the patients with threatened preterm delivery. 

## Materials and methods

This study conducted in Imam Khomaini and Razi hospitals at Ahvaz Jondishapur University of Medical Sciences (AJUMS), Iran in 2002. The ethics committees of AJUMS approved this study. The patients had given written, informed consent before enrolling in this study. This study is a randomized clinical trial. Eligible women with preterm labor between 24-37 week gestations were selected for the study. Inclusion criteria were nulliparous and multiparous pregnancies with intact membranes, showing clinical signs of preterm labor. The diagnosis of preterm labor is based on the presence of 4 uterine contractions or more over 30 minutes, each lasting at least 30 seconds, and documented cervical change (dilatation of 0-4 cm and effacement of at least 50%) (12). 

Exclusions criteria were women with clinical intrauterine infection, cervical dilatation *>*5 cm, medical complications with tocolysis like severe preeclampsia, lethal fetal anomalies, chorioamnionitis, significant antepartum hemorrhage, maternal cardiac or liver diseases, and evidence of no reassuring fetal status. In this study one hundred preterm women between 24-37 week gestations were randomly selected. In the first step all patients were hydrated by 500 ml of Ringer solutions and bed rest. Patients with gestational age lower than 34 weak took dexamethasone for fetal lung maturity. 

Patients were selected randomly to receive either oral nifedipine (Aboureihan pharmaceutical Company: Adalat, Nifedipine) or intravenous magnesium sulfate. Nifedipine tocolysis was initiated with a 10 mg capsule which was repeated every 20 min (up to a maximal dose of 30 mg during the ﬁrst hour of treatment) and then nifedipine maintenance dose was 10 mg every six hours. Tocolysis with magnesium sulfate was initiated with 10g (I.V) and then 5g (I.M) every 4 hours. In all patients, fetal heart rate, blood pressure, pulse rate, and uterine contractions were recorded. 

All patients were checked for successful prolongation of pregnancy who had not been delivered at 48 hours (primary tocolytic effects) and at more than 7days (secondary tocolytic effects) after beginning the treatment and side effects of tocolysis. Side effects were assessed with particular emphasis on hypotension, tachycardia, palpitation, flushing, headaches, dizziness, and nausea related to nifedipine side effects; and flushing, nausea, headache, drowsiness, blurred vision and respiratory and motor depression of the neonate related to magnesium sulfate side effects. If contractions did not subside, other tocolytic medication, such as isoxsuprine or indomethacin, was added (treatment failure). 

**Statistical analysis**

A statistical analysis program (SPSS version15) was used for data analysis. All characteristics and outcome variables were evaluated with percentage of them. Differences between groups analyzed by using the Mann-Whitney U test, the unpaired t student test.

## Results

**Figure 1:**
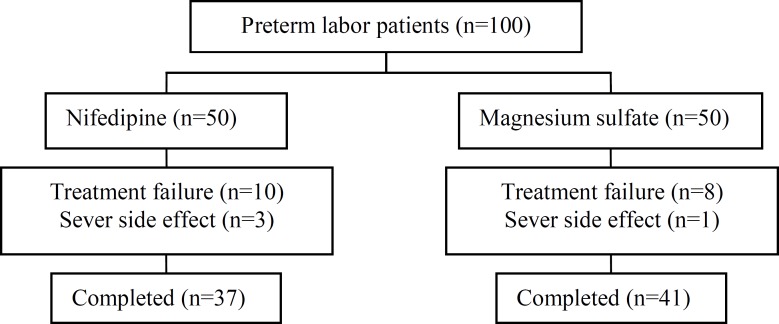
Flowchart of study

To evaluate the efficacy and safety of magnesium sulfate and nifedipine (Adalat), a total of 100 women were enrolled; 50 patients were randomly assigned to the nifedipine group and 50 were randomly assigned to the magnesium sulfate group (Figure 1). The baseline characteristics such as maternal age, parous, gestation age, prior preterm birth, abortion, twin gestations, urinary infection and hemoglobin were checked in both groups. There were no statistically signiﬁcant diﬀerences between them (Table I). On the other hand, the main outcome variables such as days gain in utero, success rate and side effects were examined in the two groups.

Two patients (4%) after 24 hours, 4 patients (8%) after 48 hours, 3 patients (6%) after 72 hours and 28 patients (56%) after 7 days had delivery in the nifedipine group and 5 patients (10%) after 24 hours, 2 patients (4%) after 48 hours, 2 patients (4%) after 72 hours and 32 patients (64%) after 7 days had delivery in the magnesium sulfate group. This characteristic was not statistically different between the two groups. In this study, 10 patients (20%) in nifedipine group and 8 patient (16%) in magnesium sulfate group had a failure treatment (contractions did not subside) and needed to take other tocolytic medications. This characteristic was also not statistically different between the two groups (Table II). 

Three patients (6%) in the nifedipine group had severe hypotension and one patient (2%) in the magnesium sulphate group had severe flushing. These side effects caused drug discontinuation. Patients in the nifedipine group and magnesium sulfate group had the general side effects: 4 cases (8%) of headache and 1 case (2%) of flushing, respectively. All of obstetric characteristics were also not statistically different (Table II).

**Table I:** Maternal and preterm labor characteristics

		**Nifedipine** **N (%)**	**Magnesium sulfate** **N (%)**	**p**-**value**
Maternal age (years)				
	<18	4 (8)	2 (4)	0.51
	18-40	43 (86)	46 (92)	0.50
	>40	3 (6)	2 (4)	0.54
Primiparous		27 (54)	24 (48)	0.50
Multiparous		23 (46)	26 (52)	0.50
Gestational age				
	<34	31 (62)	29 (58)	0.50
	>34	19 (38)	21 (42)	0.50
Prior preterm birth		2 (4)	1 (2)	0.54
Abortion		4 (8)	6 (12)	0.51
Twin gestations		2 (4)	1 (2)	0.57
UTI		7 (14)	5 (10)	0.51
Hb				
	<10 mg/dl	4 (8)	3 (6)	0.53
	<11mg/dl	6 (12)	5 (10)	0.52

UTI: urinary tube infection.

Hb: hemoglobin.

Analyzed by using the Mann-Whitney test

**Table II:** Obstetric characteristics

			**Nifedipine** **N (%)**	**Magnesium sulfate** **N (%)**	**p-value**
Delivery					
	After 24h		2 (4)	5 (10)	0.48
	After 48h		4 (8)	2 (4)	0.51
	After 72h		3 (6)	2 (4)	0.54
	After 7 days		28 (56)	32 (64)	0.50
Treatment failure			10 (20)	8 (16)	0.50
Severe side effect			3 (6)	1 (2)	0.44

Analyzed by using the Mann-Whitney test.

## Discussion

Preterm labor is a common obstetric problem that in it delivery occurs between 24 and 37 weeks before completed gestation. Prevention and treatment of preterm labor are important by reducing adverse events for the neonate. A wide range of tocolytics have been tried, but obstetricians still do not have an ideal drug available. However magnesium sulfate is the most widely used tocolytic, an effective role of it has never been established. Nifedipine is an effective and rather safe alternative tocolytic agent for management of preterm labor. We undertook this study to compare the efficacy and safety of magnesium sulfate and nifedipine in the management of preterm labor. 

In this study, 8% if patients in nifedipine group and 4% of patients in magnesium sulfate group delivered in the first 48 hours. There was no significant difference between two groups. 56% of patients in the nifedipine group and 64% of patients in the magnesium sulfate group delivery for more than 7 days. This characteristic was also not statistically different between two groups. These results have been shown by other studies. In a randomized study, one hundred ninety-two patients were enrolled. This study showed there were no differences in delivery within 48 hours in two groups (11). Another study showed two groups postponed delivery for more than 48 hours (13). 

In our study, in 6% of patients in the nifedipine group and 2% of patients in the magnesium sulfate group, therapy was discontinued because of severe side effects like hypotension and flushing. These obstetric characteristics were not statistically different. On the other hand, 20% and 16% patients in the nifedipine and magnesium sulfate group had a failure treatment because contractions did not subside and needed to take other tocolytic medications. This characteristic was also not statistically different between two groups. The same results were also obtained from the other study (13).

In a study, nifedipine compared with magnesium sulfate and ritodrine hydrochloride in the management of preterm labor. They concluded that side effects were much more in the magnesium sulfate and ritodrine group than the nifedipine group and nifedipine is an effective, safe, and well-tolerated tocolytic agent (14). In another study, Larmon and colleagues compared oral nicardipine (closely related to nifedipine) and magnesium sulfate in acute therapy for preterm labor. They showed there was a significant decrease in the time to uterine quiescence in the nicardipine group. Patients in the magnesium sulfate group had more side-effects in the form of nausea and vomiting and they were more likely to have another tocolytic agent (15).

Several investigators demonstrated that nifedipine treatment did not influence either fetal or uteroplacental circulation (16, 17). It is generally considered to be safe for both mother and fetus and it reduces respiratory distress syndrome, necrotizing enter colitis and intraventricular hemorrhages. The direct maternal adverse effects are related to the vasodilatation caused by nifedipine and are primarily headache and facial flushes. Generally, these complaints disappear within 24 hours. 

On the other hand, other factors that have contributed to the growing interest in nifedipine as a tocolytic are the availability of a wide range of immediately acting and extended-release preparations for oral use and the fact that it is very cheap. Magnesium must be used by only the infusion route and requires special monitoring and close observation. Patients taking magnesium sulfate should be monitored for toxic side effects such as respiratory depression or even cardiac arrest. Magnesium crosses the placenta and can cause respiratory and motor depression of the neonate. Moreover, Grimes and colleagues showed that the risk of total pediatric mortality was significantly higher for infants exposed to magnesium sulfate and it should not be used for tocolysis (7).

## Conclusion

In conclusion, our data in this study showed that oral nifedipine is a suitable alternative for magnesium sulfate with the same efficacy and side effects in the management of preterm labor. However, other workers have advocated that before nifedipine is introduced into clinical practice, the effectiveness should be assessed in a placebo-controlled trial and nifedipine may not be effective for all patients.
